# The complete mitochondrial genome of *Dendrolimus kikuchii* (Lepidoptera: Lasiocampidae)

**DOI:** 10.1080/23802359.2017.1365654

**Published:** 2017-08-18

**Authors:** Yu-Heng Wu, Xing-Shi Gu, Jin Xue, Xing Wang

**Affiliations:** a College of Plant Protection, Hunan Agricultural University, Changsha, China;; b Hunan Provincial Key Laboratory for Biology and Control of Plant Diseases and Insect Pests, Hunan Agricultural University, Changsha, China

**Keywords:** *Dendrolimus kikuchii* Matsumura, mitochondrial genome, evolutionary relationships

## Abstract

As a serious forest pest on coniferous trees, *Dendrolimus kikuchii* has caused widespread concern in China. Here, its complete mitochondrial genome (mitogenome) has been sequenced with 15,382 bp in length. The mitogenome has a base composition of A (40.87%), T (37.83%), C (13.43%), and G (7.87%), and consists of 13 protein-coding genes (PCGs), 22 transfer RNA (tRNA) genes, two ribosomal RNA (rRNA) genes, and an A + T-rich region. The phylogenetic relationships among the lasiocampid species were (*Trabala vishnou*+ ((*Apatelopteryx phenax*+* Euthrix laeta*) + (*Dendrolimus kikuchii*+ (*D. spectabilis*+ (*D. tabulaeformis + D. punctatus*))))), which were supported by a posterior probability of 1.00 and a high bootstrap value of 100%.

As a destructive forest pest, simao pine moth *Dendrolimus kikuchii* Matsumura, 1927 (Lepidoptera: Lasiocampidae) has an extensive range across southern China and Vietnam (Kishida and Wang 2011). Its larvae attack various coniferous trees by feeding on conifer needles, and affect the pines’ growth rate even resulting in tree death (Men et al. 2017). In September 2015, the adult of *D. Kikuchii* was collected from Shennonggu National Forest Park (26°18′00″ N, 113°56′30″ E, 900 m at attitude), Yanling county, Hunan province, China. Genomic DNA was extracted from its thorax muscles using a Wizard Genomic DNA Purification Kit and stored in Hunan Agricultural University for sequencing. Primers reported by Gu et al. (2016) were used to amplify the complete mitogenome. The fragments were proof-read and assembled by the program Geneious 8.12 (Kearse et al. 2012), and the automatic annotation by the online-program MITOS (http://mitos.bioinf.uni-leipzaig.de) (Bernt et al. 2013). The complete mitogenomes of six lasiocampid species as ingroups and two drepanid species as outgroups were obtained from NCBI. The conserved regions of the putative amino acids from all 13 protein-coding genes (PCGs) excluding the stop codons were filtrated by the software Gblock 0.91b with default settings. The phylogenetic tree was reconstructed using Bayesian inference (BI) by running over 10,000,000 generations and by maximum likelihood (ML) with 1000 replications.

The entire mitogenome of *D. kikuchii* is a closed circular molecule with 15,382 bp in length (GenBank accession number MF100138), and a base composition of A (40.87%), T (37.83%), C (13.43%), and G (7.87%). It encoded 37 genes consisting of 13 PCGs, 22 transfer RNA (tRNA) genes, and two ribosomal RNA (rRNA) genes, as well as contains a putative A + T-rich region. Almost all of the PCGs started with ATN except *cox1* with CGA. Additionally, three PCGs (*cox1*, *cox2*, *nad4*) have a single stop codon T, and the other 10 PCGs have the complete stop codon TAA. A + T-rich region is located between *rrnS1* and *trnM* with 320 bp in length and has a high AT content of 92.19%. A conserved structure consisting of the motif ‘ATAGA’ was present in the downstream 21 bp of *rrnS1*, and a microsatellite ‘(AT)_7_’ was located at the 99 bp upstream of *trnM*.

The evolutionary relationships among the lasiocampid species were reconstructed, and the topological structures of the BI and ML trees were identical (Figure 1). The Lasiocampidae species were strongly supported as a monophyletic clade by the posterior probability of 1.00 and the bootstrap value of 100%, and the relationship within this family was *Trabala*+ ((*Apatelopteryx*+* Euthrix*)+*Dendrolimus*). Furthermore, the phylogenetic position of *D. kikuchii* among the genus *Dendrolimus* was *D. kikuchii*+ (*D. spectabilis*+ (*D. tabulaeformis + D. punctatus*)), which was supported as a monophyletic clade by a posterior probability of 1.00 and a bootstrap value of 100%. The evolutionary relationships of these analysed species are consistent with previously reported results (Kononov et al. 2016; Min et al. 2016; Wu et al. 2016a, 2016b). The newly determined mitogenome is useful for understanding the evolution of the pine moths.

**Figure 1.**
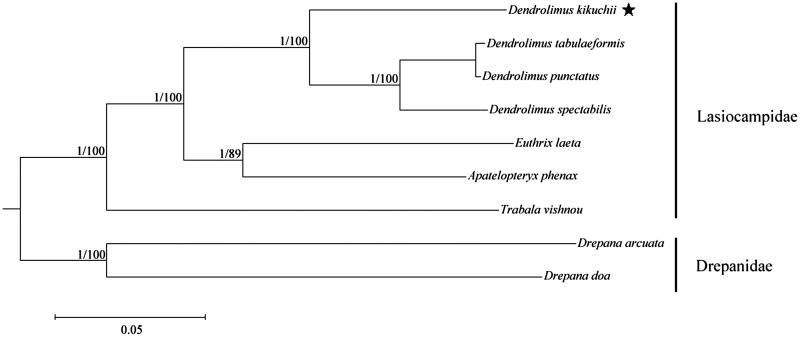
Bayesian inference and maximum likelihood phylogram constructed using 13 PCGs of mitogenomes with partitioned models. Numbers above each node indicate the ML bootstrap support values and the BI posterior probability. All the species’ accession numbers in this study are listed as below: *Apatelopteryx phenax* KJ508055, *D. kikuchii* MF100138, *Dendrolimus punctatus* NC_027156, *Dendrolimus spectabilis* NC_025763, *Dendrolimus tabulaeformis* NC_027157, *Drepana arcuata* KJ508053, *Drepana doa* KJ508058, *Euthrix laeta* NC_031507, *Trabala vishnou* KU884483.
